# Australian Notes

**Published:** 1919-11-08

**Authors:** 


### AUSTRALIAN NOTES.

FROM OUR CORRESPONDENT.

### Hospitals Association Scheme.

A SCHEME for the formation of a Metropolitan Hospitals Association has been placed before the (State) Treasurer (Mr. McPherson) by Mr. W. J. G. Turner, Secretary for the Austin Hospital for Incurables. Such an Association, it is thought, would be as beneficial for the town hospitals as it has proved for the country institutions.

### Military Nurses' Privileges.

UNDER the new housing scheme of the Repatriation Department, a military nurse of the Commonwealth enjoys the same privileges as a soldier. If she has been on active service she may purchase, without any deposit, house or land to the value of £700. On occupation she will pay a weekly rent fixed at a rate to return interest on capital and property. She is allowed thirty-seven years to pay off a brick house, and twenty years for a wooden one. Returned nurses wishing to establish themselves in business may obtain up to £50 for the purpose from the Assistance Section of the Repatriation Department.

### Tribunal for Doctors.

DR. J. LEON JONA, of Melbourne, makes the interesting proposal that a tribunal consisting exclusively of medical practitioners be constituted to control the manner in which doctors discharge their responsibilities to the public. He remarks: "At the present time, apparently, any registered medical practitioner who has sufficient conceit, self-confidence, or effrontery can set himself up as a specialist in any branch of medicine or surgery, irrespective of his personal or professional qualifications. If an independent tribunal, composed wholly of medical men, were appointed to inquire into every death reported, a great good would be done."

The *Medical Journal* remarks: "It is eminently desirable that a better control should be kept over the profession."

### The Board of Health.

The epidemic of pneumonic influenza which has swept over the whole of Australia has revealed all the shortcomings of divided control—State and Federal—and the ensuing confusion has caused much suffering and loss. But if the struggle with the epidemic revealed anything, it fully illuminated the futility, the uselessness of the present Board of Health, which has so often asked that it might be either ended or mended. Early in the epidemic the Medical Influenza Advisory Committee was formed, and among the Committee's recommendations is the need of complete reorganisation of the whole Health Department.

